# Prescription opioid use in Israel – the tide has risen, but it’s not a tidal wave

**DOI:** 10.1186/s13584-024-00648-2

**Published:** 2024-10-10

**Authors:** Yehuda Neumark, Paola Rosca

**Affiliations:** 1 Braun School of Public Health & Community Medicine, Faculty of Medicine, The Hebrew University of Jerusalem, P.O. Box 12272, Jerusalem, 9112102 Israel; 2 Department for the Treatment of Substance Abuse, Ministry of Health; The Hebrew University of Jerusalem, Jerusalem, Israel

**Keywords:** Opioid prescription use, Opioids, OUD, Israel, Policy, Fentanyl, Public health

## Abstract

The devastating human and financial costs of the ongoing global opioid crisis underscore the need for comprehensive public health strategies, effective treatment programs, and robust policy interventions to mitigate its impact. Regarding Israel, numerous reports highlight a steady increase since 2000 in prescription opioid use and the shift to more potent opioids particularly fentanyl, particularly among more marginalized population groups. In response to growing concern in the country about the rise in prescription opioid use and the consequential risk of opioid use disorder, the Israeli government, together with the country’s health service providers, implemented a series of measures to monitor and regulate opioid prescriptions and balance the need for effective pain management with the prevention of opioid abuse and its associated harms. A national opioid data monitoring system is being established, alongside the provision of addiction training for health professionals, the integration of treatment services for opioid use disorder into the nationalized primary healthcare system, and the expansion of harm reduction strategies to mitigate the health risks associated with opioid use. Additional funding for opioid-related research, and for the broader fields of addictions and mental health, is vital. In conclusion, the sum of the evidence suggests that Israel is not facing an “opioid crisis” Continued commitment, resources, and innovative approaches will be crucial to ensure that the rising tide of opioid use in Israel, particularly during and in the aftermath of the ongoing war, will not become a tidal wave.

Prior to the 1990s, pain was largely a neglected issue in patient care. Opioids, if prescribed, were mainly for short-term pain relief or for end-of-life therapy. In 1995, the American Pain Society promoted the concept of pain being a “fifth vital sign”, calling for more aggressive use of opioids for pain management. In that same year OxyContin (manufactured by Purdue Pharma) received FDA approval, and a perfect storm was created.

The ongoing “opioid crisis”, that has since plagued North America in particular, refers to the widespread medicinal and non-medicinal use of opioids - a class of drugs that includes heroin, fentanyl, and prescription pain relievers such as oxycodone and morphine. The crisis has been characterized by high rates of opioid use disorder (OUD), overdose deaths, and associated social and economic problems. Although difficult to estimate, over 16 million people worldwide are thought to be affected by OUD [1], and 2-4% of the population in the United States [2]. Over 100,000 fatal opioid overdoes occur annually worldwide [3].

The financial costs of the opioid crisis are no less staggering. The annual costs associated with OUD and fatal opioid overdoses in the United States are estimated to be over $1 trillion [4, 5]. In Israel, the mean monthly healthcare costs for adults with OUD is more than five times higher than persons without OUD, and much of these costs are associated with prescription opioids [6].

Paramount among the many and interacting factors contributing to the crisis, predominantly in the early years, was over-prescription of opioids for pain management due to misconception of safety of chronic opioid therapy and incentives for increasing volume of prescribed patients. Oxycontin sales, for example, increased in the United States from 300,000 prescriptions in 1996 to 6 million in 2001 [7]. Other key contributors include hospital discharge practices that resulted in patients using medications longer than necessary, and insufficient access to addiction treatment and to non-opioid pain treatment [8–10]. In the EU, only about half of high-risk opioid users receive opioid agonist treatment [11], and the treatment gap is even greater in the United States [12].

In Israel, as in many other countries, opioids, including oxycodone, morphine, and fentanyl, are commonly prescribed for pain management, particularly for chronic pain and cancer-related pain. Likewise, as in other countries, there is growing concern in Israel about the misuse of prescription opioids and the subsequent risk of OUD. Several reports, including some recently published in this journal, point to a steep and steady increase in Israel since 2000 in prescription opioid use – in terms of dosages and the number of patients using opioids [9, 13–19]. Much of this increase has been fueled by a rise in fentanyl and oxycodone consumption. The steepest increases were seen in low socio-economic groups, immigrant groups, and in the periphery of the country, which is generally more disadvantaged than the non-periphery [6, 19].

Among opioid-receiving members of the Maccabi Healthcare Services (Israel’s 2nd largest HMO), average daily consumption more than doubled from 4.6 MME^1^in 2010 to 10.5 MME in 2020, while the number of patients who filled a prescription increased by 15% - from 23.9/1000 to 27.6/1000 [19]. The percent of Clalit Health Services (CHS, Israel’s largest HMO) members who filled at least one opioid prescription doubled between 2010 (2.1%) and 2020 (4.2%)^2^, and in the same period, MME/capita rose by 142% and MME/prescription by 49% [18]. The number of non-oncology patients receiving high doses (≥ 90 daily MME) increased more than six-fold – from 0.3 to 1.3% of opioid-filling patients, and in 2020, one-third of the total dispensed MME was prescribed to this small group of patients. Fentanyl accounted for 82% of the total MME dispensed to these patients. Among CHS cancer patients, however, only a small annual increase in the percent who filled at least one prescription was noted between 2007 and 2011, followed by levelling-off from 2011 until 2018 [22]. The percentage of cancer patients who purchased opioids for more than 13 months similarly remained unchanged. Yet, the annual per capita OME nearly doubled from 753 OME in 2007 to 1432 in 2018.

The above cited reports do not include clinical parameters that can inform regarding the extent to which the individual dosages, and the temporal increases, were clinically justified. Without such data, the suggested conclusion of a need to “curb” prescribing opioids may be unfounded. Some maintain that these increases actually reflect improved pain management that was previously characterized by under-treatment, rather than an impending, or existing “opioid crisis” in Israel [9, 13, 22].

Indeed, indicators of opioid-related outcomes have not demonstrated parallel increases. The rates of opioid-related hospitalizations have remained stable, and in fact decreased by 49% from 2015 to 2020, while this same period saw an 85% increase in opioid procurement and 162% increase in transdermal fentanyl procurement [17]. Only a modest increase was noted in the number of persons receiving opioid agonist maintenance treatment^3^for OUD in the period 2013–2020, mainly related to persons who developed dependence from opioid medications and not to heroin or other street opioids [9, 24]. Stable rates of detoxification treatment and hospitalizations despite increases in opioid use may reflect stigma towards treatment among persons in need of treatment. The development of addiction treatment options within the HMOs may improve referral to treatment and treatment attendance (see below regarding the mental health services reform underway in Israel).

Furthermore, Israel did not experience an increase in opioid-related mortality [9], as was evidenced in North America where opioid-related deaths skyrocketed. In 2021 alone, opioid overdose deaths in the US were responsible for 3 million years of life lost and a reduction 0.65 years in the national life expectancy [25]. In Israel, opioid-related mortality actually declined from 2005 (1.3/1000 persons) to 2017 (0.19/1000) [26, 27], and subsequently remained unchanged until 2021 [17]. The low opioid-related mortality may reflect, in part, the lower prevalence of drug use and OUD in Israel, although it is generally assumed that drug-related and overdose deaths are under-reported [28]. This is due to possible missed diagnoses, misclassification of underlying causes of death, post-mortem toxicology screening that is not mandatory and does not identify fentanyl or oxycodone, and the low autopsy-rate in Israel [17, 26, 29]. As services develop within the HMOs, data regarding profiles of prescription opioid users may provide a more accurate picture of the opioid-related mortality in the country.

In a recently published report in this journal, Cohen and colleagues [29] examined the association between prescription opioid use and mortality among CHS adult non-oncological opioid patients. The likelihood of death over the 10-year period 2011–2020 was 2.23-fold and 2.37-fold higher among those receiving 50–90 MME/day and > 90 MME/day, respectively, compared with lower-dose patients (< 50 MME/day). When compared with mortality rates in the general population, the adjusted mortality hazard ratios ranged from 5.4 to 10.7 among women and 1.2–5.4 among men, depending on dose.

Numerous strategies have been employed in the United States and elsewhere to mitigate the effects of the crisis including prescription drug monitoring programs, medication management systems, improved access to pain and addiction services, health workforce education, and expanding the use of naloxone, an overdose-reversal drug^4^. The recently updated CDC Clinical Practice Guideline for Prescribing Opioids for Pain [30] address the areas of: initiating opioids for pain, opioid selection and dosage, duration and follow-up, risk assessment and mitigation. By way of example, the guidelines recommend maximizing non-pharmacologic and non-opioid treatments and considering opioid-therapy only if anticipated benefits outweigh the risks; enhancing person-centered decision-making and clinician-patient communication regarding benefits and risks, treatment goals, and therapy discontinuation strategy if benefits do not outweigh risks; clinicians should offer or arrange evidence-based treatments for patients with opioid use disorder.

Despite these efforts, the crisis continues, highlighting the critical challenge in drug policy: interventions such as supply reduction, that target one aspect of the complex issue, may lead to unintended consequences [31]. For example, in the United States, opioid-related deaths have continued to rise in the last decade, while at the same time opioid-prescribing declined. Deaths from fentanyl alone tripled from 2016 to 2021 reaching > 80,000 in 2021 [32], and the synthetic analog of fentanyl, carfentanyl − 10,000 times more potent than morphine and 100 times more potent than fentanyl, has been responsible for many overdose outbreaks in the United States [33]. As the “iron law of prohibition” (should have) taught us, regulations to reduce opioid prescribing may lead to a shift to illicit opioid use, increases in injection-related infectious diseases, and an increase in overdose deaths [31, 34]. Israel’s law enforcement agencies will need to continue strengthening and adapting their strategies to address the challenges posed by a potential rise in illicit opioid use.

Since 2015, the Israeli government has implemented numerous measures to monitor and regulate opioid prescriptions and balance the need for effective pain management with the prevention of opioid abuse and its associated harms. Some of these measures include electronic prescription systems, guidelines for physicians on the appropriate use of opioids, and education and awareness campaigns. Addiction training courses for health professionals have been initiated, and this should become an essential part of all health professional education. Health professionals also need to be better skilled in communicating with their patients about the potential risks and benefits of opioid treatment, and alternative therapy options [35]. Frameworks and modalities aimed at treating OUD have been established, but more are needed. Easily accessible and non-stigmatic services for the early detection and treatment of OUD have been integrated into primary care pain clinics in the past few years, and are undergoing expansion. Israel also employs harm reduction strategies to minimize the health risks associated with opioid use, and these need to be expanded including, perhaps, the establishment of supervised consumption sites [36, 37].

A special committee of the Ministry of Health was established in 2019 to advise on policies to reduce prescription opioid use and misuse. The committee recognized the urgent need for data monitoring which will allow identification of changes in prescription practices, emerging trends, and age groups at high risk, and assess the effectiveness of measures undertaken to reduce prescription opioid use. The first step taken by the Ministry, therefore, was to request data from the four HMOs on opioid prescriptions (in OME units) twice a year by age group, dosage, and length of treatment, in order to create a comprehensive national database for opioid usage data. In 2022, the Ministry instructed the HMOs and hospitals to develop institutional strategies to optimize prescription opioid oversight (the first recommendation for physicians listed in the box below). The Bureau of National Security has been recently chosen by the Israeli Parliament to create a National Focal Point that will aggregate comprehensive information from relevant government ministries and agencies. Availability of precise and timely data will guide the development and adoption of evidence-based policies.

Some of the 50 recently published draft recommendations ranked by the committee members appear in the box below.

**Table:** 

For Physicians: Tightening oversight of prescriptions (including pre-authorization of fentanyl patch prescriptions, allow digital opioid prescriptions only), and enhance knowledge about referral of patients to rehab services. Other recommendations, such as reassessment of opioid-using patients prior to prescription renewal, and changing death certificate notification to require opioid-use to appear as cause of death, were ranked lower by the committee.
For Pharmacists: Update the Dangerous Drugs Ordinance (to include fentanyl and buprenorphine); mandatory training of pharmacists; automated notification of opioid purchase (including from private pharmacies) in patient’s HMO file; more detailed explanations to opioid-using patients. Other recommendations, such as integrating clinical pharmacists in the care and follow-up of opioid-using patients, and restricting patient’s purchasing to a single pharmacy, were ranked lower by the committee.
For Nurses: Provide instruction about opioid use to patients prior to release from hospital; organize instructional events for nursing school staff and students.
For Institutional Administration: Establish national committees in each HMO to address the opioid phenomenon; establish committees in all hospitals to monitor use and prescribing patterns and establish guidelines for appropriate opioid use; integrate opioid use indicators in the Israel National Program for Quality Indicators in Community Healthcare. The recommendation to establish multi-professional pain/drug rehab clinics was ranked lower by the committee.
For Regulatory Practices: Establish quality indicators for opioid use in the Israel National Program for Quality Indicators in Community Healthcare; establish continuity of care guidelines for patients transitioning from hospital to primary care; establish a national computerized system to monitor the supply, prescription and purchase of opioids; regulatory monitoring of high-prescribing physicians. Other regulatory recommendations such as a ban on contributions by pharmaceutical companies to medical institutions, and imposing restrictions on opioid users (e.g., temporary driving license suspension) were ranked low by the committee.

The country’s nationalized health system and fully electronic patient health records put Israel in a unique position to implement effective monitoring, regulation, and treatment strategies to address the opioid challenge more effectively than other countries.

Indeed, some of the Ministry’s recommendations have been, and are being implemented by the HMOs. In 2022, CHS began requiring a specialist’s authorization for fentanyl initiation. In the year following introduction of that policy, fentanyl prescription initiations decreased steeply by 81% among non-cancer patients, compared to the previous year [38]. The decrease was more pronounced among non-elderly patients, perhaps due to the curtailing of unwarranted prescriptions in the younger age groups, and/or raised awareness of the potential risks associated with fentanyl use among younger people. Introduction of the specialist’s authorization policy was also followed by a more modest drop in total opioid MME, and a slight increase in non-fentanyl opioid MME [39]. This is the first indication that tighter prescription regulation may be effective in curbing prescription opioid use in Israel.

Israel is also on the verge of a major reform in the provision of addiction treatment services that are currently under the supervision and subsidization of the Ministry of Health, rather than being part of the services provided by the HMOs. This fragmentation seriously impairs vital continuity of care and treatment outcomes [39]. In 2022, a special committee was established to decide whether medical addiction treatment services, now delivered by the Ministry of Health, should be transferred to the HMOs. The committee concluded that the HMOs are not yet ready to administer these services, and the government approved a three-year NIS 85 million (US$22 million) plan which will allow the health funds to prepare for the reform and develop and scale-up necessary services. These include early detection and treatment in primary care through implementation of the Screening, Brief Intervention, and Referral to Treatment (SBIRT) model [40], establishment of out-patient and day-care clinics for the treatment of OUD patients with chronic pain, patients with co-occurring disorders (dual-diagnosis), and behavioral addictions (particularly gambling), and establishment of alternatives to hospitalization of co-occurring disorder patients, such as Soteria-like intensive residential services [41]. This healthcare reform poses a major challenge for the Israeli health system; however, the potential benefits of an integrated care model make it an important goal for improving addiction treatment and overall patient care.

## Conclusions

The opioid crisis is a global public health problem, and Israel is not exempt from its impact. Serious concerns have been expressed about the misuse of prescription opioids and the shift to more potent opioids and the consequences thereof. Yet, despite increases in opioid use, and a widely cited claim that “Israel became the country with the highest consumption of prescription opioids in the world” [42], the sum of the evidence suggests that Israel is not facing an “opioid crisis”.

The Israeli government and healthcare providers have begun to implement measures and policies to mitigate risk among existing and new opioid users. It is expected however, that it will take time, perhaps even a few years, for the benefits of the proposed and already-initiated policies - in particular a reduction in opioid misuse and morality, to become evident, given the time needed for policy implementation and adoption, and for changes to occur in clinical practice. There are also unanticipated events which impact negatively on the mental wellbeing of the population leading to increased demand for pain and anxiety relieving medications.

In this regard, it is important to note the October 7th attack on Israel by Hamas, which has had significant and wide-reaching impacts on various aspects of Israeli society. While the full impact of the October 7th attack and its ongoing aftermath on opioid use in Israel will become clearer over time, a steep increase in psychopathology [43] and in prescription opioid use is already evident [44–46], similar to the surge in opioid use in the United States following 9/11. The steepest rises in opioids consumption have been noted among those living nearer to the border with Gaza, Arabs and women [46]. The need for pain-relief among the large number of victims of war-related injuries is also contributing to the rising tide of prescription opioid use. As the Ministry of Defense is funding medical treatment of veterans who receive compensation, it will be important to link data concerning opioid prescription with data from the HMOs and the Ministry of Health, to prevent misuse and double-dispensing.

Higher rates of OUD, overdose, and fatalities have persistently been observed in socioeconomically marginalized group [47, 48], underscoring the need for targeted primary prevention interventions and policies that address the social determinants of health in efforts to combat the opioid crisis [3].

So, as Israel takes steps towards reforming its addiction treatment system, and while we all wait for safer and more effective pain-management medications to replace opioid analgesics for chronic pain and shift to non-pharmacological interventions, a “whole-of-society and whole-of-government approach” [49, 50] must be adopted, that will ensure that patients who genuinely need opioids for pain management and other conditions have access to them, while minimizing the risk of misuse and broader societal harms. This is especially urgent in light of the ongoing war and the resultant national and personal traumas.

## Data Availability

Not applicable.

## Abbreviations

CDC: Centers for Disease Control and Prevention
CHS: Clalit Health Services
CM: Contingency management
DDD: Defined daily dose
EU: European Union
FDA: U. S. Food and Drug Administration
HMO: Health maintenance organization
MME: Morphine milligram equivalent
OME: Oral morphine equivalent
OUD: Opioid use disorder
SBIRT: Screening, Brief Intervention, and Referral to Treatment

## Footnotes

1 In the context of pharmacoepidemiology, two standardizing measures are commonly used when analyzing trends in prescription opioid use – DDD (Defined Daily Dose) and MME (Morphine Milligram Equivalent) [or OME (Oral Morphine Equivalents)] [20–22]. While complementary, these two measures serve distinct roles in understanding and managing opioid use, and do not produce identical results, especially when the data includes opioids of varying potency. Briefly, DDD is the assumed average maintenance dose per day for a drug used for its main indication in adults. DDDs are not always reflective of the actual dose prescribed, especially for opioids where individual dosing can vary widely based on pain severity, patient tolerance, and other factors. OME is used to quantify and compare the potency of different opioids by converting them into an equivalent dose of oral morphine, providing a clinically relevant way to assess and compare opioid potency, and measure the total opioid load a patient is receiving. Nearly all recent analyses of Israeli opioid prescription trends, use MME or OME.

2 In their analysis of CHS patients, Miron and colleagues (2021) [16] estimated the proportion of Clalit Health Service patients who filled an opioid prescription each year between 2014 and 2018 to be 10% - considerably higher than the 4.2% reported by Dressler et al. [18]. The discrepancy can be explained by the inclusion of codeine (the most commonly prescribed opioid) in the analyses conducted by Miron et al., but not in the report by Dressler et al.

3 Medication-assisted treatment [including buprenorphine, methadone or naltrexone, with add-on psychotherapy, overdose education, naloxone distribution, and contingency management (CM)], particularly methadone + CM, has been shown to yield significant savings in costs related to OUD morbidity and mortality, when compared with no treatment [23].

4 In March 2023, the FDA approved naloxone nasal spray “Narcan” as the first over-the-counter drug to reverse fentanyl overdoses. Naloxone has been available without prescription for many years in some countries, although the effectiveness of this policy is yet to be fully assessed [3]. The nasal spray antidote Nyxoid^®^ (Mundipharma) has been registered in Israel but has yet to be included in the national healthcare basket.
